# The Effects of Occupational and Leisure Time Physical Activity on Health-Related Quality of Life: A Repeated-Measures Longitudinal Study

**DOI:** 10.1007/s40279-025-02382-4

**Published:** 2026-01-10

**Authors:** Stephanie A. Prince, Tyler Thomas, Aviroop Biswas

**Affiliations:** 1 Centre for Surveillance Applied Research, Public Health Agency of Canada, 785 Carling Avenue, Ottawa, ON KA1 0K9 Canada; 2 School of Epidemiology and Public Health, Faculty of Medicine, University of Ottawa, Ottawa, Canada; 3 Public Health Sciences, Queen’s University, Kingston, Canada; 4 Institute for Work & Health, Toronto, Canada; 5 Dalla Lana School of Public Health, University of Toronto, Toronto, Canada

## Abstract

**Background:**

High leisure time physical activity (LTPA) is consistently linked to health benefits, whereas high occupational physical activity (OPA) has been associated with adverse health outcomes, a phenomenon known as the “physical activity health paradox.” This study examined how OPA and LTPA interact to influence health-related quality of life (HRQL), a measure of physical and mental well-being.

**Methods:**

A repeated-measures longitudinal study was conducted using data from 7382 Canadian workers (aged 18–75 years) in the National Population Health Survey (1994–2011). Multilevel growth curve models were used to assess associations between self-reported measures of LTPA (active, inactive), OPA (sit, walk/light loads, heavy loads), and OLTPA, a composite variable of their combined effects, with the Health Utility Index score (HRQL), adjusting for sociodemographic and health covariates.

**Results:**

Active LTPA was associated with better HRQL, whereas OPA was not. Results suggested a curvilinear response between OLTPA and HRQL with age whereby middle-aged workers lifting heavy loads at work and active LTPA have lower HRQL than older workers in the same group. Findings were similar among males, but among older females (60 + years) who lifted heavy loads at work, being inactive rather than active in leisure resulted in higher HRQL.

**Conclusion:**

The association between LTPA, OPA, and HRQL is complex, varying across age and sex. These findings highlight the need for physical activity recommendations that are sensitive to occupational demands when promoting health and well-being.

**Supplementary Information:**

The online version contains supplementary material available at 10.1007/s40279-025-02382-4.

## Key Findings/Implications

**Table:** 

Evidence from this repeated-measures longitudinal analysis in a sample of Canadian workers suggests that leisure-time physical activity, but not occupational physical activity, confers benefits for maintaining health-related quality of life.
Workers who mostly sat at work and who were inactive in leisure time had the lowest health-related quality of life over time regardless of sex.
As workers age, engaging in sufficient leisure-time physical activity contributes to better health-related quality of life, underscoring the importance of promoting leisure-time physical activity for all workers, including those in physically demanding jobs.

## Introduction

Regular physical activity is recognized as important for achieving and maintaining good physical and mental health [1, 2]. However, emerging evidence indicates that different types of physical activity may have varying effects on health. Meta-analytic evidence based on several large cohort studies indicates that high occupational physical activity (OPA), especially from physically strenuous work, shows inconsistent associations with health [3, 4]. Some studies have shown that OPA may provide little-to-no health benefits and can even be associated with adverse health outcomes such as a greater risk for cardiovascular disease, type 2 diabetes, osteoarthritis, and premature death [3–6]. Furthermore, a systematic review and an individual-participant meta-analysis showed that, for several health outcomes, including all-cause mortality and cardiovascular disease, higher levels of leisure time physical activity (LTPA) are consistently protective among workers with low levels of OPA but confer less protection among those with moderate or high levels of OPA [7, 8]. Yet, the World Health Organization guidelines on physical activity and sedentary behavior do not distinguish between LTPA and OPA for health benefits, highlighting the need for more evidence on the differing health effects of OPA [9].

Occupations characterized by high OPA can include tasks that are performed for prolonged periods of time without sufficient opportunity for rest such as standing, lifting, or carrying heavy loads, as well as awkward postures. In the USA, approximately 39% of the civilian workforce perform physically demanding job tasks [10], with similar levels in Canada, particularly among workers in lower skilled (e.g., nurse aides, food and beverage servers) and labor-oriented jobs (e.g., fruit pickers, cleaning staff) [11]. In addition, workers with higher OPA have been shown to perform less LTPA compared with workers in more sedentary jobs [12], which have been linked to a need for recovery from the fatigue and pain associated with physically demanding jobs as barriers [13].

Most research comparing OPA and LTPA has relied on cohorts with a single baseline exposure assessment linked to long-term outcomes such as mortality or cardiovascular disease incidence [4, 8, 14]. Few studies have collected repeated measures on OPA and LTPA to strengthen causal inference and assess proximal health outcomes that directly reflect the effects of these exposures. In addition, there have been criticisms of insufficient adjustment for socioeconomic factors (e.g., income, education, occupation) and smoking, raising concerns that the observed paradoxical effects of OPA on health outcomes may be at least partly attributable to residual confounding [15, 16]. Importantly, the association between LTPA and health may also be confounded by socioeconomic factors [17].

Health-related quality of life (HRQL) is a widely used measure of the overall physical and mental well-being and functional status of a population. HRQL is highly responsive to changes in health status over time [18], and poor HRQL has been shown to be predictive of increased healthcare utilization, morbidity, and premature mortality [19–22]. Furthermore, HRQL incorporates the subjective experiences of individuals, providing insights into how health affects daily life that mortality or morbidity data alone cannot capture. This makes HRQL an important policy-relevant indicator of population health. Studies have consistently shown that higher physical activity levels can improve HRQL in adults [23]. However, previous work has largely explored the independent effects of LTPA in relation to HRQL, with few studies examining the effects of OPA [23, 24], while, to the best of our knowledge, no studies have explored the combined effects of LTPA and OPA on HRQL.

Both HRQL and total physical activity (including LTPA and OPA) are known to decline with age [25–27]. This is important given that the workforce in Canada, like most industrialized countries, is aging, with participation among older Canadians (55 + years), especially women, growing [28]. Aging workers experience natural age-related declines in their fitness, which may impact their capacity to meet the physical demands of work [29–31]. Compounding this concern, fitness among working age adults, especially blue-collar workers, has also been declining [32, 33]. Understanding how the association between OPA, LTPA, and HRQL differs with age is important to inform targeted interventions.

Research also indicates that HRQL trajectories differ by sex [25], and the effects of high OPA on health may also be sex-specific, with males experiencing worse outcomes [3]. Gender differences in occupational roles further complicate this picture, as men and women may be disproportionately represented across occupations, perform different tasks within the same occupations, and perceive occupational demands differently [34, 35]. Evidence also suggests that the protective effects of the same volume of physical activity on health outcomes may differ between sexes [36]. However, limited research has examined sex differences in the OPA–LTPA interaction on health. To date, most of the evidence, particularly for higher intensities of OPA, have been derived from male-only samples or studies underpowered to detect sex-specific effects [3, 8].

The primary objective of this study was to examine the combined effects of OPA and LTPA on HRQL among working adults in Canada. The secondary objective was to understand the potential moderating role of age and sex on the associations between OPA/LTPA and HRQL. We hypothesized that workers with low OPA and active LTPA would result in better HRQL, whereas workers with high OPA and inactive LTPA would have worse HRQL.

## Methods

### Data Sources

#### National Population Health Survey (NPHS)

The NPHS was a population health survey that collected both cross-sectional and longitudinal data related to health status, health-care utilization, and health determinants from a representative sample of most people living in Canada [37]. The survey excluded populations residing on Indigenous Reserves, those residing on Canadian Forces Bases, the territories, and residents of certain remote regions in Quebec and Ontario. All survey responses were collected through computer-assisted interviewing that took place over the telephone or in person. This analysis used the longitudinal NPHS data, which followed participants from the 1994/1995 baseline cohort who were reinterviewed every 2 years across all nine available NPHS cycles (1994/1995 to 2010/2011). The NPHS experienced modest annual attrition due to nonresponse, resulting in a cumulative nonresponse rate of 8.1% over the full panel [38].

### Study Inclusion Criteria

The study included all respondents who were between the ages of 18 and 75 years and who reported working at least part-time for pay or profit, had at least two cycles of data, and had complete data on LTPA, OPA, and covariates.

### Independent Variables (Time Varying)

#### Occupational Physical Activity (OPA)

OPA was assessed by self-reported usual daily activities or work habits. This variable asked participants to recall their previous 3 months and choose the category that best described their usual daily activities or work habits including: (1) “usually sit and don’t walk very much”; (2) “stand or walk quite a lot”; (3) “usually lift or carry light loads”; or( 4) “do heavy work or carry very heavy loads.” For the purpose of this study, OPA was recategorized into three groups to include those who: (1) usually sit (category 1) [referent]; (2) walk or light loads (combining categories 2 and 3: “stand or walk quite a lot” and “usually lift or carry light loads”); and (3) heavy loads (category 4).

#### Leisure-Time Physical Activity (LTPA)

LTPA was assessed by self-reported leisure physical activities using an adaptation of the Minnesota Leisure-Time Physical Activity Questionnaire [39]. Participants were asked to recall their participation in leisure activities in the previous 3 months (including frequency and duration) from a list of 20 common activities. A physical activity index was created based on the calculated energy expenditure of the leisure activities [40]. The physical activity index categorized respondents as being (1) active (≥ 3 kcal/kg/day or 180 MET-min/day), (2) moderately active (1.5–2.9 kcal/kg/day or 90–174 MET-min/day), or (3) inactive (referent; < 1.5 kcal/kg/day or < 90 MET-min/day). For the purpose of this study, LTPA was recategorized into two groups: active (combined categories 1 and 2: active and moderately active) and inactive (category 3) [referent].

#### Occupational–Leisure Time Physical Activity Interaction (OLTPA)

A composite predictor variable (OLTPA) was created using the combination of OPA and LTPA. The composite variable OLTPA included the following categories: (1) sit at work/inactive leisure (referent); (2) sit at work/active leisure; (3) walk or light loads/inactive leisure; (4) walk or light loads/active leisure; (5) heavy loads/inactive leisure; and (6) heavy loads/active leisure.

Face validity of the OPA measure was compared with a self-reported single item question on physical exertion measured on a 5-point Likert scale (“Does your job require a lot of physical effort?”) in the NPHS work stress module that was adapted from Karasek and Theorell’s Job Content Questionnaire [41] (Supplementary Table 1). Physical exertion scores increased with increasing OPA levels consistently for both sexes. In addition, the scores were consistent within OPA categories regardless of LTPA level.

### Dependent Variables (Time Varying)

#### Health-Related Quality of Life (HRQL)

HRQL was assessed by the Health Utility Index Mark 3 (HUI); a generic measure of health status that synthesizes both the quantitative and qualitative aspects of health, and is a strong predictor of future mortality [19, 42]. It is a composite index that evaluates eight attributes of overall functional health: hearing, vision, speech, mobility, dexterity, cognition (memory and thinking), emotion (feelings), and pain and discomfort. The index summary values range from − 0.36 to 1.00. On the scale, the most preferred health level is rated as 1.00 and death is rated as 0.00; negative scores reflect health states considered worse than death as derived from societal preferences [42]. A change of 0.03 in the HUI score is considered the smallest clinically meaningful difference [18]. Statistics Canada uses HUI as a health indicator for computing health-adjusted life expectancy [43]. Given the skewed nature of the HUI variable, we applied an arcsine-based transformation (HUIARC) developed by Statistics Canada [44] to improve model fit, defined as: HUIARC = arcsine((2 × (HUI + 0.36)/1.36) − 1).

### Covariates

 All covariates were self-reported and included age (centered around the baseline mean = 36.1 years), sex (male, female), body mass index (BMI; kg/m^2^), alcohol consumption (regular (> once per month), occasional (< once per month), former (no drink in past 12 months), abstainer (never drank in lifetime)), education level (< secondary, secondary graduate, other post-secondary, college or university degree), household income adequacy (quintiles considers household income relative to the number of people living in the household), smoking status (daily smoker, occasional smoker, former smoker, never smoked), continuous hours worked at primary job, shift work, number of children under 12 years in the household, and work stress (score based on 12-item index measured on a 5-point Likert scale [45]). All confounders were included as time-varying variables, except for age and sex, which were included as responses at baseline and chosen on the basis of their availability and known association with both OPA/LTPA and HRQL [46–48]. Supplementary Fig. 1 provides a directed acyclic graph (DAG) showing causal relationships, confounders, and effect modifiers considered in the analysis. A measure of fruit and vegetable consumption was considered but was available only for cycles 5–9 and was, therefore, not included in the models. Work stress was available in cycles 1 and 4–9; if participants had two or more work stress responses, missing values (as is the case for cycles 2 and 3) were imputed on the basis of average work stress scores.

### Statistical Analyses

All analyses were conducted using R Studio v 4.4.1 [49]. Descriptive statistics for the sample characteristics at baseline were examined using means and percentages and 95% confidence intervals (CIs). To account for the complex sampling design of the NPHS, population weights were applied to all analyses. The 500 bootstrap weights provided by Statistics Canada were used for variance estimation of descriptive statistics. A *p*-value of 0.01 was used to reduce the potential for type 1 error. To be retained in the analyses, respondents had to have complete data on OPA, LTPA, HUI, and all covariates (except for work stress as outline above) at each included time point.

Multilevel growth curve models were used to estimate the longitudinal association between OLTPA and HUI with observations over time (level 1) nested within an individual (level 2). Thus, time-varying variables (e.g., OLTPA) were included at level 1 and time-invariant variables (e.g., sex) at level 2. Two-level models were built to predict HUI associated with OLTPA with a random intercept and random slope for time. This allowed us to properly capture the correlation between observations of the same person, as well as the heterogeneity of HUI trends between participants. Participants who did not participate in all cycles of the survey remained in the sample but contributed less data than others. A person-period dataset (one record per participant) was created for each cycle in which OLTPA and HUI were available. Models were fit using the R package nlme.

The primary models were nonlinear and able to capture the curvature in the group trends with age. A quadratic fit allowed us to explore any nonlinearity, similar to other work by Statistics Canada [44]. We tested both quadratic and cubic fits similarly, and found the quadratic fit to be sufficient, as the cubic model indicated overfitting. The key improvement of the quadratic model over a linear model is the back-transformed HUI scores, which are plotted for visual interpretation. The linear model’s back-transformed HUI scores did not show clinically meaningful separation between groups (HUI score < 0.3); however, the quadratic fit provided several interesting and clinically meaningful differences. These findings are in-line with previous analyses by Statistics Canada [44], who also reported nonlinear trends in their modelling of HUI using the NPHS. The quadratic growth model was specified as: HUIarc_ij_ = *β*_0_ + *β*_1_AGE_*ij*_ + *β*_2_OLTPA_*ij*_ + *β*_3_OLTPA_*ij*_ × AGE_*ij*_ + *β*_4_SEX_*i*_ + *β*_5_INCOME_*ij*_ + *β*_6_EDUCATION_*ij*_ + *β*_7_CHILDREN_*ij*_ + *β*_8_WSTRESS_*ij*_ + *β*_9_WHOURS_*ij*_ + *β*_10_SHIFT_*ij*_ + *β*_11_ALCOHOL_*ij*_ + *β*_12_SMOKE_*ij*_ + β_13_BMI_*ij*_ + β 14*(*AGE_*ij*_)^2^ + *β*_15_OLTPA_*ij*_ × (AGE_*ij*_)^2^ + (*ζ*_0*i*_ + *ζ*_1*i*_AGE_*ij*_ + *ε*_*ij*_). In the quadratic age models, age groups are based on visual interpretation of the graphs.

The linear model specification was identical to the quadratic models, but replaced AGE with TIME and added baseline-centered age as a time-invariant covariate. Linear models included: (1) an unadjusted model with interaction terms between OPA/LTPA/OLTPA and time; (2) an adjusted model with sociodemographic confounders (sex, age, household income, education level, number of children in the household under 12 years); (3) a model with the addition of work-related confounders (hours worked, shift work, work stress); and (4) a model with the addition of health and behavioral confounders (alcohol consumption, smoking status, BMI). This graded approach allowed us to assess the change in magnitude/effect of the work/leisure activity categories after adjusting for sets of confounders (all else the same). The final linear growth model was specified as: HUIarc_*ij*_ = *β*_0_ + *β*_1_TIME_*ij*_ + *β*_2_OLTPA_*ij*_ + *β*_3_OLTPA_*ij*_ × TIME_*ij*_ + *β*_4_SEX_*i*_ + *β*_5_AGE*_*C_*i*_ + *β*_6_INCOME_*ij*_ + *β*_7_EDUCATION_*ij*_ + *β*_8_CHILDREN_*ij*_ + *β*_9_WSTRESS_*ij*_ + *β*_10_WHOURS_*ij*_ + *β*_11_SHIFT_*ij*_ + *β*_12_ALCOHOL_*ij*_ + *β*_13_SMOKE_*ij*_ + *β*_14_BMI_*ij*_ + (*ζ*_0*i*_ + *ζ*_1*i*_TIME_*ij*_ + *ε*_*ij*_).

## Results

**Fig. 1:**
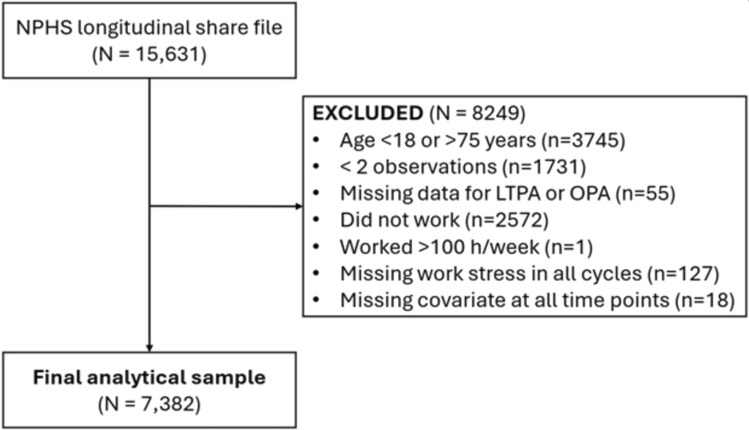
Participant flow chart

A total of 7,382 participants from the NPHS longitudinal share file were retained for analysis (Fig. 1). Table 1 provides the weighted baseline descriptives for the sample. At baseline, the average age was 36.3 years with a slightly greater representation of males (53.7%) than females (46.3%). Most participants had post-secondary education (58.8%) and mid-to-upper income levels (88.8%). The average number of hours worked per week was 38.0, with men working more on average than women (42.3 h/week for men compared with 33.1 h/week for women). Most participants (77%) reported working regular shifts. Most reported that they stood, walked a lot, or usually lifted or carried light loads at work (69.3%), and most were inactive in their leisure time (60%). The average HUI score was 0.91, suggesting most had mild or no disability [50]. This value was higher than the average score reported for Canadian adults aged 40 + years in the 1994/95 NPHS (0.83) [25].

**Table 1:** Baseline characteristics

Characteristics	Unweighted sample size	Weighted mean or % (95% CI)		
		Total	Males	Females
**Age, years**	7382	36.3 (36.0, 36.5)	36.8 (36.4, 37.1)	35.7 (35.3, 36.1)
**Sex, %**				
Male	3668	53.7 (52.8, 54.6)	–	–
Female	3714	46.3 (45.4, 47.2)	–	–
**Education level, %**				
Less than secondary	1176	14.9 (13.8, 15.9)	17.5 (16.0, 19.1)	11.8 (10.4, 13.2)
Secondary grad	1202	16.7 (15.6, 17.8)	16.2 (14.6, 17.8)	17.2 (15.6, 18.8)
Other post-secondary	2133	28.8 (27.5, 30.1)	26.8 (24.9, 28.7)	31.1 (29.1, 33.1)
College or university degree	2871	40.0 (38.2, 41.0)	39.4 (37.4, 41.5)	39.8 (37.8, 41.9)
**Household income adequacy quintile, %**				
Lowest	320	3.2 (2.7, 3.7)	2.6 (1.9, 3.3)	3.9 (3.2, 4.6)
Low-mid	667	8.0 (7.1, 8.8)	7.6 (6.4, 8.9)	8.3 (7.1, 9.5)
Middle	2035	25.4 (24.0, 26.8)	24.3 (22.4, 26.2)	26.7 (25.0, 28.5)
Upper-mid	3147	43.1 (41.5, 44.7)	43.5 (41.4, 45.7)	42.6 (40.4, 44.7)
Highest	1213	20.3 (19.1, 21.6)	21.9 (20.1, 23.7)	18.5 (16.8, 20.2)
**Children < 12 years in the household, #**	7382	0.63 (59.8, 65.7)	0.59 (0.55, 0.63)	0.67 (0.63, 0.71)
**Alcohol consumption, %**				
Regular drinker	4871	66.9 (65.4, 68.3)	76.0 (74.1, 78.0)	56.2 (54.1, 58.3)
Occasional drinker	1531	19.4 (18.2, 20.6)	12.4 (10.9, 13.9)	27.6 (25.7, 29.5)
Former drinker	678	8.7 (7.9, 9.5)	7.3 (6.2, 8.3)	10.3 (9.0, 11.6)
Abstainer	302	5.0 (4.2, 5.8)	4.4 (3.3, 5.4)	5.9 (4.7, 7.1)
**Smoking status, %**				
Daily smoker	2161	27.4 (26.2, 28.6)	28.5 (26.8, 30.3)	26.0 (24.2, 27.8)
Occasional smoker	392	5.5 (4.8, 6.3)	5.7 (4.6, 6.8)	5.3 (4.4, 6.2)
Former smoker	2171	29.5 (28.2, 30.7)	30.8 (28.9, 32.6)	28.0 (26.1, 29.7)
Never smoked	2658	37.6 (36.2, 39.1)	35.0 (33.1, 36.8)	40.8 (38.7, 42.8)
**Body mass index, kg/m**^**2**^	7382	25.2 (25.0, 25.3)	25.9 (25.8, 26.1)	24.3 (24.1, 24.5)
**Work hours, hours/week**	7382	38.0 (37.6, 38.5)	42.3 (41.7, 42.9)	33.1 (32.5, 33.6)
Work stress, score*	7382	19.3 (19.2, 19.5)	18.8 (18.6, 19.1)	19.9 (19.7, 20.2)
**Shift work, %**				
Regular shift (day/night/eve)	5649	77.4 (76.1, 78.7)	76.5 (74.8, 78.3)	78.4 (76.5, 80.2)
Rotating or split shift	813	10.3 (9.5, 11.2)	11.4 (10.1, 12.7)	9.1 (8.0, 10.2)
Irregular/on-call/other	920	12.3 (11.2, 13.3)	12.0 (10.6, 13.5)	12.5 (11.1, 14.0)
**Health Utility Index, score**	7382	0.91 (0.90, 0.91)	0.91 (0.90, 0.92)	0.91 (0.90, 0.91)
**OPA level, %**				
Sitting	1518	21.6 (20.3, 22.8)	20.9 (19.0, 22.8)	22.3 (20.5, 24.1)
Walk/light loads	5154	69.3 (67.9, 70.6)	65.0 (62.9, 67.0)	74.2 (72.3, 76.2)
Heavy loads	710	9.2 (8.3, 10.1)	14.1 (12.7, 15.5)	3.5 (2.7, 4.3)
**LTPA level, %**				
Active	2998	40.0 (38.0, 41.2)	42.4 (40.2, 44.6)	36.3 (34.1, 38.5)
Inactive	4384	60.0 (58.8, 62.0)	57.6 (55.4, 59.8)	63.7 (61.5, 65.9)

*LTPA* leisure time physical activity, *OPA* occupational physical activity, *Missing work stress scores were imputed based on the average across all available cycles

Table 2 presents the distribution of the sample across OLTPA categories. The largest combination group comprised workers who walked/lifted light loads at work and were inactive in leisure (41.0%), with a higher proportion of females than males in this category (46.1% versus 36.5%). The smallest group was those who reported lifting heavy loads at work and being active in leisure (3.4%), with more males than females in this category (5.4% versus 1.2%).

**Table 2:** Sample distribution across OLTPA categories

	Unweighted sample size	Weighted % (95% CI)		
		Total	Males	Females
**OLTPA categories**				
Sit at work / inactive in leisure	952	13.7 (12.6, 14.7)	12.3 (10.7, 13.9)	15.2 (13.7, 16.7)
Sit at work / active in leisure	566	7.9 (7.0, 8.8)	8.6 (7.3, 9.9)	7.1 (6.0, 8.1)
Walk/light loads at work / inactive in leisure	2985	41.0 (40.0, 42.5)	36.5 (34.5, 38.5)	46.1 (43.9, 48.4)
Walk/light loads at work / active in leisure	2169	28.3 (26.9, 29.7)	28.4 (26.5, 30.4)	28.1 (26.0, 30.1)
Heavy loads at work / inactive in leisure	447	5.8 (5.1, 6.4)	8.8 (7.7, 9.8)	2.3 (1.6, 3.0)
Heavy loads at work / active in leisure	263	3.4 (2.8, 4.0)	5.4 (4.3, 6.4)	1.2 (0.7, 1.6)

*OLTPA* occupational–leisure time physical activity combination

Secondary linear time models (Supplementary Figs. 2–4 and Supplementary Tables 2–4) found significant main effects for LTPA whereby being active compared with inactive was associated with greater HRQL at baseline. No significant main effects were observed for OPA. Sex-stratified linear results suggested similar main effects from LTPA but reduced effect estimates in females. Higher OPA was significantly and positively associated with HRQL in males, but with negative associations in females. Examining the combined effects of OPA and LTPA (i.e., OLTPA), compared with sit at work/inactive in leisure (referent), sit at work/active in leisure and walk and light loads/inactive in leisure had significant main effects suggesting they had lower HRQL at baseline. Workers lifting heavy loads and active in leisure had positive main effects, suggesting this group had the highest HRQL at baseline. There was also a significant time-by-OLTPA interaction, whereby, compared with sitting at work and inactive in leisure, all other groups, except those lifting heavy loads and active in leisure had higher HRQL over time. Those sitting at work and inactive in leisure had the lowest HRQL over time.

**Fig. 2:**
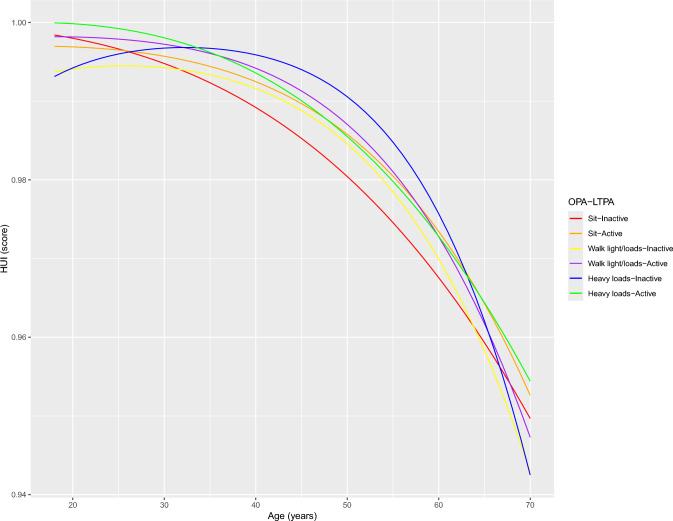
Quadratic age model for back-transformed HUI trends across OLTPA groups in the full sample. Results derived from a quadratic age model fit with random intercept and slope (fully adjusted model, holding all other variables constant)

Quadratic age models suggest that the associations between OLTPA and HRQL are not linear and vary with age (Fig. 2). Younger workers (~ 18–35 years) generally reported higher HRQL compared with older workers. In addition, among younger workers, those lifting heavy loads but inactive in leisure had the lowest HRQL, whereas those lifting heavy loads and active had the highest HRQL. In middle age (~ 35–60 years), the heavy loads–inactive group had the highest HRQL while the sitters–inactive had the lowest. All active workers in this age group have higher HRQL than those who engaged in sitting at work. With older age (~ 60–70 years), there is a change in the association between OLTPA and HRQL. Older workers lifting heavy loads and inactive in leisure experienced the steepest decline in HRQL, whereas those active in leisure had the highest HRQL, followed closely by those sitting at work and active in leisure.

**Fig. 3:**
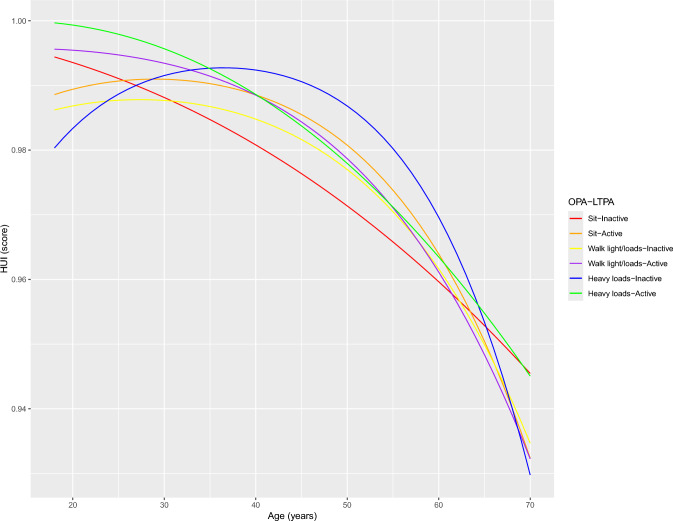
Quadratic age model for back-transformed HUI trends across OLTPA groups among males. Results derived from a quadratic age model fit with random intercept and slope (fully adjusted model, holding all other variables constant)

Sex-stratified quadratic models suggested similar results in males (Fig. 3), with those lifting heavy loads and inactive showing the steepest decline among older workers.

**Fig. 4:**
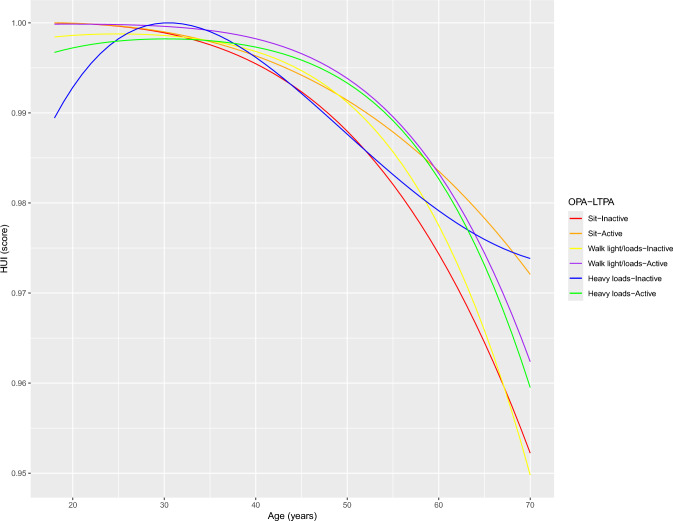
Quadratic age model for back-transformed HUI trends across OLTPA groups among females. Results derived from a quadratic age model fit with random intercept and slope (fully adjusted model, holding all other variables constant)

Among females, quadratic model results differed from males and the overall sample (Fig. 4). Younger female workers (~ 18–35 years) generally reported higher HRQL compared with older female workers. Female workers who were active in leisure had higher HRQL than those who were inactive, regardless of OPA. After about age 35, female workers sitting at work and inactive in leisure generally had the lowest levels of HRQL. With older age (~ 60 + years), female workers sitting at work but active in leisure had the highest HRQL. In older age, females who lifted heavy loads at work but were inactive in leisure had higher HRQL than those who were active at work and in leisure.

## Discussion

This study improves upon previous studies by examining the combined effects of LTPA and OPA on a proximal indicator of health (HRQL) using a longitudinal, repeated-measures design. As such, it provides important evidence that enhances our understanding of the combined role of OPA and LTPA in health. We found that being active during leisure time was associated with higher HRQL but observed no significant main effect for OPA. Results suggested a curvilinear response between OLTPA and HRQL with age, whereby middle-aged workers lifting heavy loads at work may not incur the same benefits from LTPA as older workers. Findings were similar among males, but among older females (~ 60 + years) lifting heavy loads at work, those who were inactive in leisure had higher HRQL than those who were active in leisure. Overall, for most workers, engaging in adequate LTPA was found to be important for maintaining HRQL.

### Comparisons with the Literature

Evidence supports consistent positive associations between LTPA and HRQL in the general adult population [23, 24, 51]. However, few studies have explored associations between OPA and HRQL, and most have assessed the effects of OPA among other domains of physical activity using cross-sectional data [52–54]. These studies suggest a stronger benefit from LTPA compared with OPA for HRQL, with OPA potentially providing greater benefit with older age.

Most research has also examined OPA and LTPA independently, with few studies investigating the mixed effect of physical activity domains. Recent evidence suggests that, for most workers, engaging in LTPA is important, but with greater benefit among those with more sedentary occupations. This is observed across a variety of outcomes, including many related to the dimensions of the HUI (e.g., mobility, cognition, emotion, and pain and discomfort) [7, 8, 55–58]. Our study aligns with these findings, suggesting that LTPA confers consistent benefit for HRQL among those in more sedentary occupations across the age span. However, with increasing age, LTPA becomes more important for those who engage in more strenuous activity on the job.

Review evidence across a multitude of other health outcomes suggests that both males and females generally benefit from LTPA, although research on sex differences remains limited due to few studies examining these effects [1]. Studies have found largely consistent results between males and females regarding the association between OPA, and its interaction with LTPA, and health outcomes, although some notable variations exist [7, 8]. For example, more strenuous OPA may be protective against premature all-cause mortality in women, whereas it may increase the risk among men [3, 4]. Moreover, while male workers with high OPA and moderate LTPA showed a nonsignificant decreased risk, females in the same group had a nonsignificant increased risk for cardiovascular mortality [8]. This aligns with our findings for older female workers, whereby those with strenuous OPA and who were active in leisure had lower HRQL compared with those inactive in leisure. Sex differences likely arise from a multitude of factors. It is possible that the variation with females may be due to fewer studies providing data on this subgroup, a lower prevalence of females in jobs involving physically demanding tasks, greater caregiving and household burden among females reducing the opportunity for LTPA, and the protective role of estrogen in female health [34, 59].

### Implications for Public Health

Mechanistic evidence suggests that higher OPA combined with inadequate recovery can lead to elevated heart rate, blood pressure, inflammation, and musculoskeletal pain and overuse injuries [60, 61]. Furthermore, while OPA may involve physical exertion, it may not translate to improvements in cardiorespiratory fitness that are typically associated with LTPA [60]. There is also evidence to suggest that low cardiorespiratory fitness may modify the effect of OPA on health outcomes [31]. However, studies also show that engaging in high volumes of OPA and LTPA may overload the cardiovascular system, resulting in increased risk for cardiovascular disease [62, 63].

Recent studies examining worker movement profiles have found that profiles suggestive of active leisure time, including among those with active work, generally have higher cardiorespiratory fitness, more favorable body composition, and lower cardiovascular disease risk [64–66]. Furthermore, those who are more active at work but inactive in leisure may have lower CRF and experience no reduction in cardiovascular disease risk [64, 66]. The ‘fit-for-work principle’ suggests that having adequate cardiorespiratory fitness to meet the physical demands of the job may protect against the detrimental associations between high OPA and some poor health outcomes, especially among workers from middle age (~ 45 years) onward [67–69]. Furthermore, engaging in high OPA while also having low LTPA and low cardiorespiratory fitness has been associated with the highest risk for all-cause mortality [70].

Cardiorespiratory fitness is also one of the strongest predictors of morbidity and mortality among adults and declines with age (especially after 45 years) [71, 72]. Cardiorespiratory fitness is also positively associated with HRQL [73, 74]. LTPA is an established means to improve cardiorespiratory fitness [1]. Encouraging and promoting LTPA is an important public health message for all workers, including those in physically demanding jobs who are older. Canadian workers with higher OPA tend to report less LTPA and more recreational screen time than workers with low OPA [12]. However, it is important to recognize that the physical demands of an occupation may present a barrier (due to musculoskeletal pain, fatigue, and job strain) for workers to engage in sufficient LTPA [75, 76]. Improving cardiorespiratory fitness among workers serves as a pathway for preserving HRQL in the working population. Most workplace physical activity interventions have included white-collar or desk-based workers [77], but workplace physical activity programs have shown promise for increasing fitness in those with physically demanding jobs, with mixed results for improving work ability [78]. Future work is needed to further examine the interplay of OPA and LTPA on health considering the potential for sex- and age-related effects.

### Strengths and Limitations

This study has several strengths, including the use of a nationally representative sample of Canadians, a prospective cohort study design with repeated measures on both the exposure and outcome to permit stronger causal inferences, the use of quadratic age models to assess the degree of nonlinearity in the association between OLTPA and HRQL, adjustment for important confounders previously missing from several studies (e.g., work stress, work hours, shift work, household income, and smoking), and sex- and age-stratified analyses. Most previous studies also used analytic samples of workers that were not nationally representative and assessed OPA and LTPA at one time point and follow-up (often for decades) to a distal outcome such as mortality or cardiovascular disease [14]. A recent review on OPA and health outcomes urged researchers to conduct sex-stratified analyses to provide a more thorough understanding of the health effects of OPA in males and females [4].

While this study has many strengths, one of the biggest limitations is the use of self-reported OPA and LTPA. The use of self-report methods may have introduced recall and response biases due to difficulty in recall and social desirability. Both OPA and LTPA were recalled on the basis of the previous 3 months. Longer recall periods could result in greater misclassification of physical activity, especially activities that are more prevalent [79].

While LTPA was estimated on the basis of energy expenditure using a previously validated method, OPA was measured more crudely, based on questionnaire categories. In addition, the question used to define OPA also considered usual daily activity or work habits, and it is possible that this led to a misclassification of OPA based on non-work activity, as well as due to a generalization of usual habits. We did, however, ascertain that OPA had face validity to discern between physical work demands. This makes it difficult to accurately measure the combination of posture, exertion, and movement patterns that together determine energy expenditure at work. There is also potential for substantial variability both between and within the broad OPA categories, which should be considered when interpreting results. Furthermore, different aspects of OPA may have distinct health effects. For example, sitting and standing postures have been associated with adverse outcomes [80, 81], while some lifting, pushing, or awkward postures may also be detrimental [82]. Conversely, more regular movement at work (e.g., via walking) could provide benefits.

While we statistically adjusted for important confounding factors, there is the potential for unaccounted residual confounding including diet quality and cardiorespiratory fitness. Fruit and vegetable consumption was considered but was available only for cycles 5–9. The NPHS also does not have a measure of cardiorespiratory fitness. There is also the potential for reverse causation whereby workers who experienced declines in health and HRQL may have been unable to continue in jobs with heavier OPA. Moreover, higher LTPA might enable an individual to tolerate higher OPA for longer, whereas high OPA could limit an individual’s ability to engage in LTPA. The analytical sample was relatively healthy, with an average HUI score of 0.91 (95% CI 0.90–0.91), suggesting the potential for the healthy worker effect, whereby healthier workers can remain in physically demanding work at older ages, whereas those who are less healthy or less fit may transition to lighter tasks or leave such work.

Finally, our sample size, especially of workers who lift heavy loads (and who are active in leisure), may have been too small to detect associations between OLTPA and HRQL. This is especially an issue in our sex-stratified analyses, where fewer females engaged in high OPA and active LTPA, as is often seen in other studies [8].

## Conclusions

To the best of our knowledge, this study is the first to examine the longitudinal effects of LTPA, OPA, and their combination on HRQL using repeated exposure measures. The results highlight that LTPA, but not OPA, confers benefits for maintaining HRQL. Sex-stratified results also indicate that OPA may provide a positive effect among males and a negative effect among females. Workers who mostly sit at work and who were inactive in leisure time had the lowest HRQL over time regardless of sex. Middle-aged workers lifting heavy loads may not incur the same benefits from LTPA as older workers. These findings highlight that, as workers age, engaging in sufficient LTPA contributes to better HRQL, underscoring the importance of promoting LTPA for all workers, including those in physically demanding jobs.

## Supplementary Information

Below is the link to the electronic supplementary material.

Supplementary file1 (PDF 314 KB)

Supplementary file2 (PDF 174 KB)

## Declarations

### Data availability

The datasets analyzed for this study are available through the Research Data Centers (RDC) Program at Statistics Canada (https://www.statcan.gc.ca/en/microdata/data-centres).
