# Supplementary material for: The Effects of Occupational and Leisure Time Physical Activity on Health-Related Quality of Life: A Repeated-Measures Longitudinal Study
Source: Sports Med. 2026 Jan 10;56(7):1771–84. doi: 10.1007/s40279-025-02382-4 (PMC13388346; doi:10.1007/s40279-025-02382-4)
Supplement: Supplementary file 1 — Supplementary file1 (PDF 314 KB) [file 40279_2025_2382_MOESM1_ESM.pdf]

Title: The effects of occupational and leisure time physical activity on health-related quality of life: A repeated measures longitudinal study
Journal: Sports Medicine
Authors: Stephanie A. Prince*, Tyler Thomas, Aviroop Biswas
***Corresponding author:** Centre for Surveillance and Applied Research, Public Health Agency of Canada, stephanie.prince.ware@phac-aspc.gc.ca

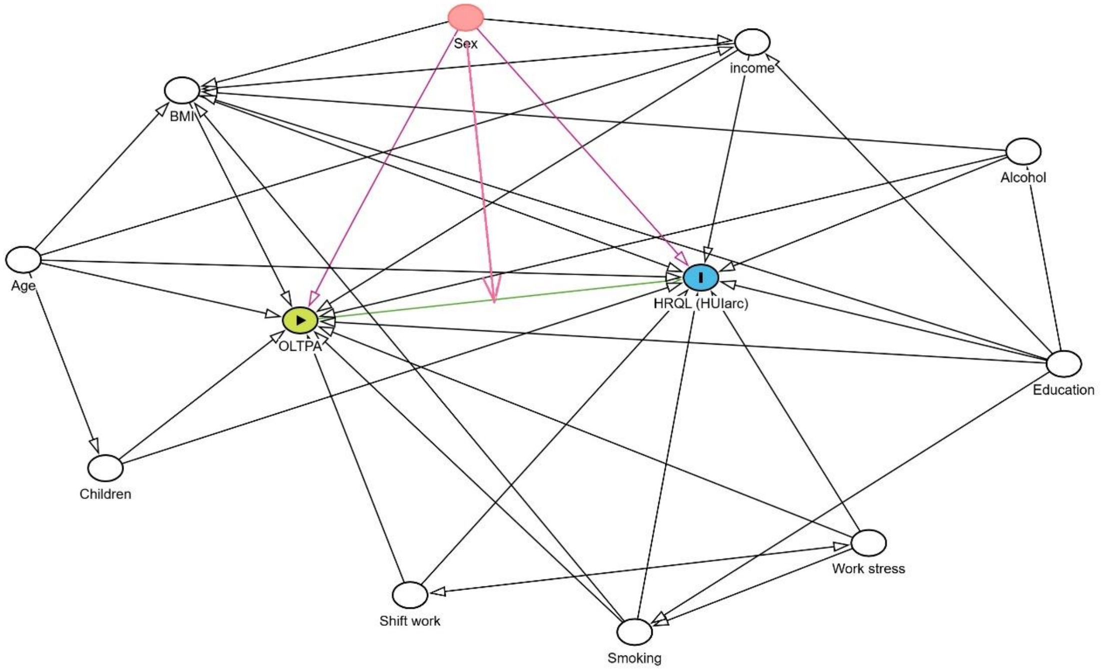

Supplementary figure 1. Directed Acyclic Graph showing relationship including causal relationships and confounders and effect modifiers considered in analysis.

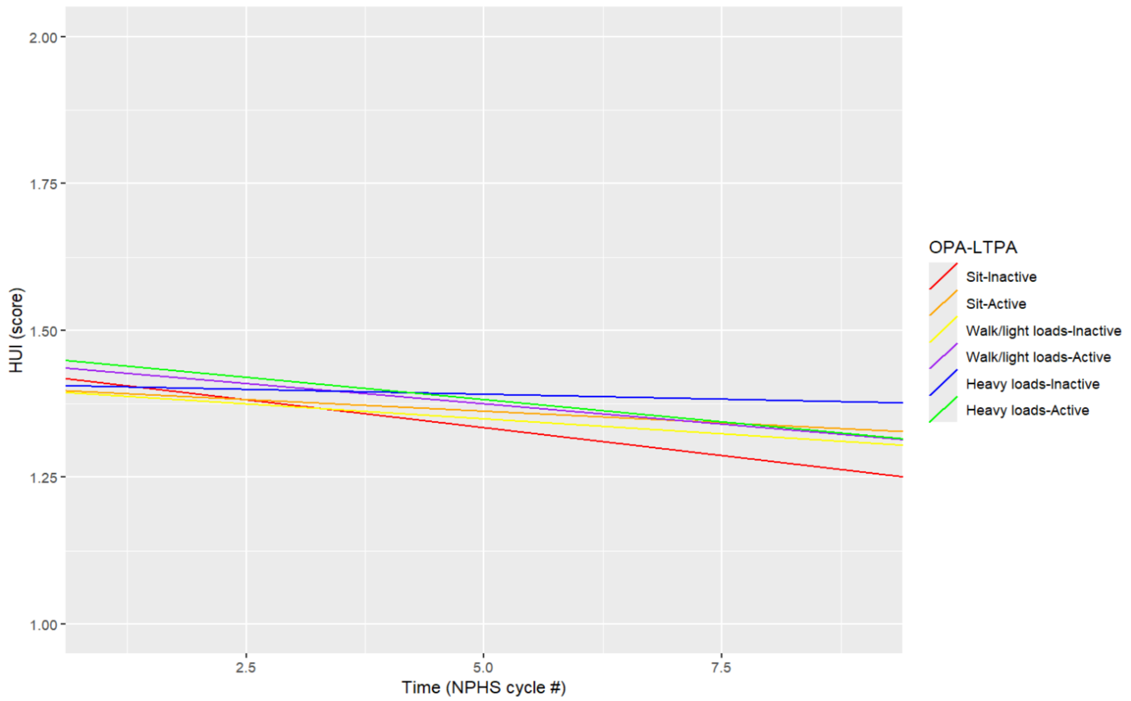

Supplementary figure 2. HUIARC trends over time for each OLTPA group. Results derived from a linear time model with arcsine transformed HUI fit with random intercept and slope (fully adjusted model, holding all other variables constant).

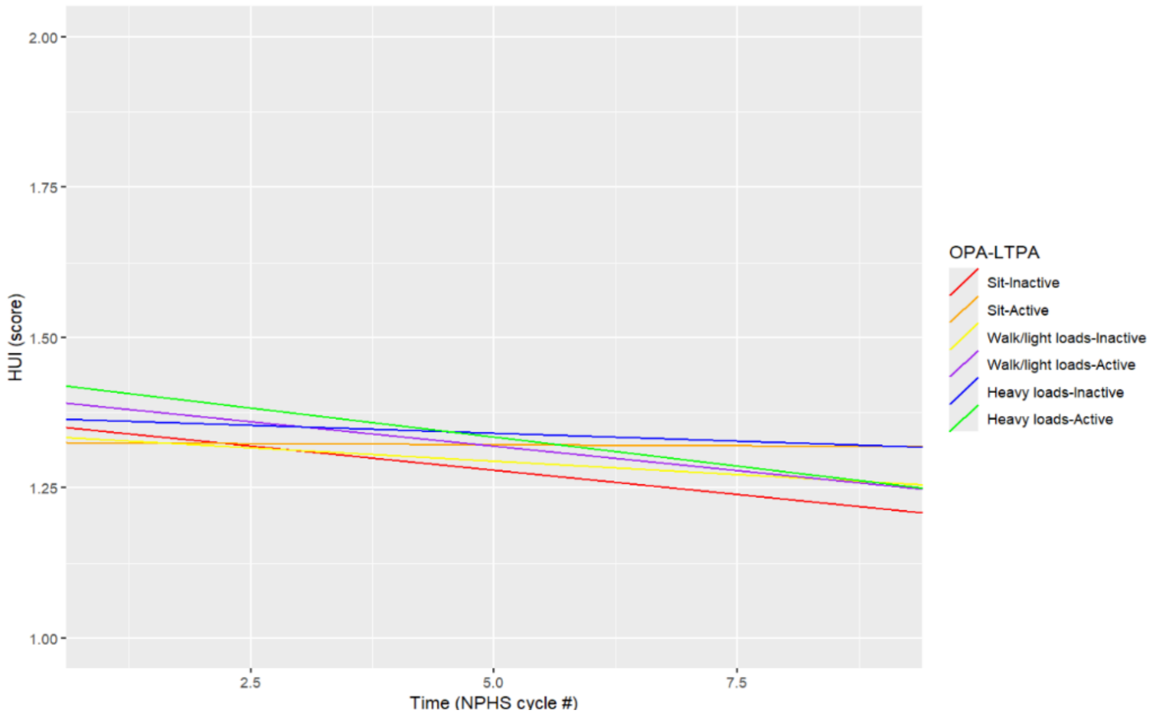

Supplementary figure 3. HUIARC trends over time for each OLTPA group among males. Results derived from a linear time model with arcsine transformed HUI fit with random intercept and slope (fully adjusted model, holding all other variables constant).

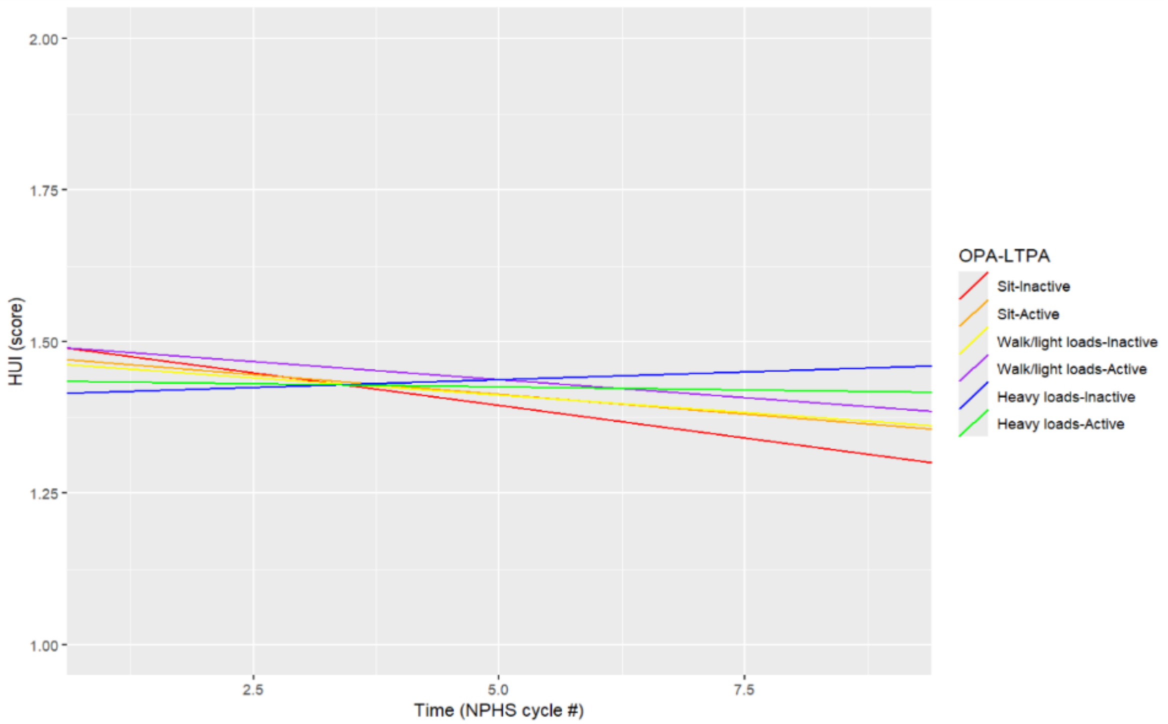

Supplementary figure 4. HUIARC trends over time for each OLTPA group among females. Results derived from a linear time model with arcsine transformed HUI fit with random intercept and slope (fully adjusted model, holding all other variables constant).
