# Supplementary material for: The Effects of Occupational and Leisure Time Physical Activity on Health-Related Quality of Life: A Repeated-Measures Longitudinal Study
Source: Sports Med. 2026 Jan 10;56(7):1771–84. doi: 10.1007/s40279-025-02382-4 (PMC13388346; doi:10.1007/s40279-025-02382-4)
Supplement: Supplementary file 2 — Supplementary file2 (PDF 174 KB) [file 40279_2025_2382_MOESM2_ESM.pdf]

Title: The effects of occupational and leisure time physical activity on health-related quality of life: A repeated measures longitudinal study
Journal: Sports Medicine
Authors: Stephanie A. Prince*, Tyler Thomas, Aviroop Biswas
***Corresponding author:** Centre for Surveillance and Applied Research, Public Health Agency of Canada, stephanie.prince.ware@phac-aspc.gc.ca

Supplementary table 1. Physical exertion scores by OPA and OLTPA categories

Characteristic	Unweighted sample size	Weighted physical exertion score (95% CI)		
		Total	Males	Females
**OPA**				
Sit	1518	1.11 (1.01, 1.21)	1.18 (1.03, 1.33)	1.04 (0.95, 1.14)
Walk/light loads	5154	2.13 (2.08, 2.19)	2.18 (2.11, 2.26)	2.08 (2.00, 2.15)
Heavy loads	710	3.55 (3.46, 3.64)	3.53 (3.44, 3.63)	3.63 (3.47, 3.78)
**OLTPA**				
Sit at work / inactive in leisure	952	1.08 (0.97, 1.20)	1.07 (0.88, 1.26)	1.10 (0.98, 1.22)
Sit at work / active in leisure	566	1.17 (0.99, 1.35)	1.35 (1.08, 1.62)	0.93 (0.78, 1.08)
Walk and light loads at work / inactive in leisure	2985	2.16 (2.09, 2.23)	2.21 (2.11, 2.31)	2.11 (2.00, 2.21)
Walk and light loads at work / active in leisure	2169	2.10 (2.02, 2.17)	2.15 (2.06, 2.25)	2.02 (1.91, 2/13)
Heavy loads at work / inactive in leisure	447	3.58 (3.48, 3.68)	3.56 (3.46, 3.67)	3.65 (3.44, 3.87)
Heavy loads at work / active in leisure	263	3.51 (3.36, 3.65)	3.49 (3.32, 3.67)	3.56 (3.33, 3.82)

OLTPA – Occupational-leisure time physical activity interaction, OPA – occupational physical activity

Supplementary table 2. Associations between OPA, LTPA and Health Utility Index scores, Males + Females (N=7382), NPHS, 1994-2011

**Physical activity exposure variables**	**Model 1. Unadjusted model**		**Model 2: Adjusted for sociodemographic covariates[a]**		**Model 3: Model 2 + adjustment for work-related covariates[b]**		**Model 4: Model 3 + adjustment for health and behaviour covariates[c]**	
	**Main effect β (95% CI)**	**Time x main effect β (95% CI)**	**Main effect β (95% CI)**	**Time x main effect β (95% CI)**	**Main effect β (95% CI)**	**Time x main effect β (95% CI)**	**Main effect β (95% CI)**	**Time x main effect β (95% CI)**
**OPA**								
Time	-0.012 (-0.015, -0.009)		-0.014 (-0.017, -0.012)		-0.015 (-0.018, -0.012)		-0.013 (-0.016, -0.011)	
Sit (REF)	-	-	-	-	-	-	-	-
Walk/light loads	-0.008 (-0.023, 0.007)	0.003 (-0.000, 0.005)	-0.003 (-0.018, 0.012)	0.002 (-0.001, 0.005)	0.001 (-0.014, 0.016)	0.002 (-0.001, 0.005)	0.002 (-0.013, 0.017)	0.002 (-0.001, 0.005)
Heavy loads	0.003 (-0.022, 0.027)	0.005 (-0.000, 0.010)	0.005 (-0.020, 0.029)	0.004 (-0.001, 0.010)	0.010 (-0.014, 0.035)	0.005 (0.000, 0.010)	0.013 (-0.011, 0.038)	0.005 (0.000, 0.010)
**LTPA**								
Time	-0.010 (-0.012, -0.008)		-0.013 (-0.015, -0.011)		-0.013 (-0.015, -0.011)		-0.012 (-0.014, -0.010)	
Inactive (REF)	-	-	-	-	-	-	-	-
Active	0.035 (0.023, 0.048)	-0.001 (-0.004, 0.001)	0.030 (0.017, 0.042)	-0.001 (-0.003, 0.002)	0.029 (0.017, 0.042)	-0.001 (-0.003, 0.002)	0.027 (0.015, 0.040)	-0.001 (-0.003, 0.002)
**Combined OPA and LTPA**								
Time	-0.017 (-0.020, -0.014)		-0.020 (-0.023, -0.016)		-0.021 (-0.024, -0.017)		-0.019 (-0.022, -0.015)	
Sit at work / inactive in leisure (REF)	-	-	-	-	-	-	-	-
Sit at work / active in leisure	-0.016 (-0.041, 0.009)	0.010 (0.005, 0.014)	-0.023 (-0.048, 0.002)	0.011 (0.006, 0.016)	-0.025 (-0.050, -0.000)	0.011 (0.007, 0.016)	-0.027 (-0.052, -0.003)	0.011 (0.006, 0.016)
Walk/light loads at work / inactive in leisure	-0.037 (-0.056, -0.018)	0.009 (0.005, 0.013)	-0.033 (-0.053, -0.014)	0.009 (0.005, 0.013)	-0.030 (-0.049, -0.011)	0.009 (0.005, 0.013)	-0.029 (-0.048, -0.009)	0.009 (0.005, 0.013)
Walk/light loads at work / active in leisure	0.012 (-0.009, 0.032)	0.005 (0.001, 0.009)	0.011 (-0.009, 0.031)	0.006 (0.002, 0.009)	0.015 (-0.006, 0.035)	0.006 (0.002, 0.009)	0.014 (-0.006, 0.034)	0.005 (0.001, 0.009)
Heavy loads at work / inactive in leisure	-0.032 (-0.063, -0.001)	0.015 (0.009, 0.022)	-0.030 (-0.061, 0.001)	0.015 (0.009, 0.021)	-0.024 (-0.054, 0.007)	0.016 (0.009, 0.022)	-0.021 (-0.052, 0.010)	0.016 (0.009, 0.022)

Heavy loads at work / active in leisure	0.030 (-0.005, 0.064)	0.002 (-0.004, 0.009)	0.024 (-0.010, 0.059)	0.003 (-0.004, 0.010)	0.026 (-0.009, 0.061)	0.004 (-0.002, 0.011)	0.028 (-0.006, 0.063)	0.004 (-0.003, 0.011)

a. **Adjusted for age (centred at baseline), sex, income adequacy, education level, number of children <12 years in the household**
b. **a + adjusted for work stress, work hours, shift type**
c. **b + adjusted for alcohol consumption, smoking status, body mass index**
Note: all models include time as a fixed effect.

Supplementary table 3. Associations between OPA, LTPA and Health Utility Index scores, Males (N=3668), NPHS, 1994-2011

Physical activity exposure variables	Model 1. Unadjusted model		Model 2: Adjusted for sociodemographic covariates[a]		Model 3: Model 2 + adjustment for work-related covariates[b]		Model 4: Model 3 + adjustment for health and behaviour covariates[c]	
	Main effect β (95% CI)	Time x main effect β (95% CI)	Main effect β (95% CI)	Time x main effect β (95% CI)	Main effect β (95% CI)	Time x main effect β (95% CI)	Main effect β (95% CI)	Time x main effect β (95% CI)
**OPA**								
Time	-0.006 (-0.010, -0.002)		-0.009 (-0.013, -0.005)		-0.009 (-0.013, -0.005)		-0.008 (-0.012, -0.004)	
Sit (REF)	-	-	-	-	-	-	-	-
Walk/light loads	0.014 (-0.008, 0.036)	-0.003 (-0.008, 0.001)	0.016 (-0.006, 0.039)	-0.003 (-0.008, 0.001)	0.020 (-0.002, 0.042)	-0.004 (-0.008, 0.001)	0.022 (0.000, 0.045)	-0.004 (-0.008, 0.000)
Heavy loads	0.041 (0.011, 0.071)	-0.003 (-0.009, 0.003)	0.042 (0.013, 0073)	-0.003 (-0.010, 0.003)	0.047 (0.017, 0.077)	-0.003 (-0.009, 0.003)	0.051 (0.021, 0.081)	-0.003 (-0.009, 0.003)
**LTPA**								
Time	-0.008 (-0.010, -0.005)		-0.010 (-0.013, -0.007)		-0.010 (-0.013, -0.007)		-0.010 (-0.013, -0.007)	
Inactive (REF)	-	-	-	-	-	-	-	-
Active	0.053 (0.036, 0.071)	-0.004 (-0.008, -0.001)	0.046 (0.028, 0.063)	-0.003 (-0.007, 0.000)	0.046 (0.029, 0.064)	-0.004 (-0.007, -0.000)	0.043 (0.026, 0.061)	-0.003 (-0.007, 0.000)
**Combined OPA and LTPA**								
Time	-0.014 (-0.019, -0.009)		-0.017 (-0.022, -0.011)		-0.017 (-0.022, -0.011)		-0.016 (-0.021, -0.011)	
Sit at work / inactive in leisure (REF)	-	-	-	-	-	-	-	-
Sit at work / active in leisure	-0.021 (-0.058, 0.016)	0.014 (0.007, 0.021)	-0.032 (-0.070, 0.005)	0.016 (0.008, 0.023)	-0.032 (-0.070, 0.005)	0.015 (0.008, 0.022)	-0.034 (-0.071, 0.003)	0.015 (0.008, 0.022)
Walk/light loads at work / inactive in leisure	-0.027 (-0.056, 0.001)	0.007 (0.002, 0.013)	-0.028 (-0.056, 0.001)	0.008 (0.002, 0.014)	-0.024 (-0.053, 0.004)	0.008 (0.002, 0.013)	-0.021 (-0.050, 0.007)	0.007 (0.001, 0.013)
Walk/light loads at work / active in leisure	0.042 (0.011, 0.072)	-0.000 (-0.006, 0.006)	0.036 (0.002, 0.066)	0.001 (-0.005, 0.007)	0.041 (0.011, 0.071)	-0.000 (-0.006, 0.006)	0.040 (0.010, 0.070)	-0.000 (-0.006, 0.006)
Heavy loads at work / inactive in leisure	-0.003 (-0.040, 0.035)	0.011 (0.003, 0.019)	-0.002 (-0.039, 0.036)	0.011 (0.003, 0.019)	0.004 (-0.033, 0.042)	0.011 (0.003, 0.019)	0.007 (-0.030, 0.045)	0.011 (0.003, 0.019)

Heavy loads at work / active in leisure	0.075 (0.033, 0.118)	-0.005 (-0.013, 0.004)	0.066 (0.023, 0.108)	-0.003 (-0.012, 0.005)	0.068 (0.026, 0.111)	-0.003 (-0.012, 0.006)	0.070 (0.028, 0.113)	-0.003 (-0.012, 0.005)

a. **Adjusted for age (centred at baseline), income adequacy, education level, number of children <12 years in the household**
b. **a + adjusted for work stress, work hours, shift type**
c. **b + adjusted for alcohol consumption, smoking status, body mass index**
Note: all models include time as a fixed effect.

Supplementary table 4. Associations between OPA, LTPA and Health Utility Index scores, Females (N=3714), NPHS, 1994-2011

Physical activity exposure variables	Model 1. Unadjusted model		Model 2: Adjusted for sociodemographic covariates[a]		Model 3: Model 2 + adjustment for work-related covariates[b]		Model 4: Model 3 + adjustment for health and behaviour covariates[c]	
	Main effect β (95% CI)	Time x main effect β (95% CI)	Main effect β (95% CI)	Time x main effect β (95% CI)	Main effect β (95% CI)	Time x main effect β (95% CI)	Main effect β (95% CI)	Time x main effect β (95% CI)
**OPA**								
Time	-0.016 (-0.020, -0.012)		-0.019 (-0.023, -0.015)		-0.020 (-0.023, -0.016)		-0.017 (-0.021, -0.013)	
Sit (REF)	-	-	-	-	-	-	-	-
Walk/light loads	-0.025 (-0.045, -0.004)	0.007 (0.003, 0.011)	-0.017 (-0.038, 0.003)	0.006 (0.002, 0.010)	-0.013 (-0.033, 0.007)	0.007 (0.003, 0.011)	-0.011 (-0.032, 0.009)	0.006 (0.002, 0.010)
Heavy loads	-0.092 (-0.140, -0.043)	0.020 (0.011, 0.029)	-0.081 (-0.130, -0.033)	0.018 (0.009, 0.028)	-0.075 (-0.123, -0.027)	0.020 (0.011, 0.029)	-0.069 (-0.117, -0.021)	0.019 (0.010, 0.029)
**LTPA**								
Time	-0.012 (-0.014, -0.009)		-0.015 (-0.018, -0.012)		-0.016 (-0.019, -0.013)		-0.014 (-0.017, -0.011)	
Inactive (REF)	-	-	-	-	-	-	-	-
Active	0.018 (0.000, 0.026)	0.001 (-0.002, 0.005)	0.015 (-0.002, 0.033)	0.002 (-0.001, 0.005)	0.014 (-0.004, 0.032)	0.002 (-0.001, 0.006)	0.013 (-0.005, 0.031)	0.002 (-0.001, 0.005)
**Combined OPA and LTPA**								
Time	-0.020 (-0.024, -0.015)		-0.023 (-0.028, -0.018)		-0.024 (-0.029, -0.019)		-0.021 (-0.026, -0.017)	
Sit at work / inactive in leisure (REF)	-	-	-	-	-	-	-	-
Sit at work / active in leisure	-0.015 (-0.049, 0.018)	0.007 (0.001, 0.014)	-0.017 (-0.051, 0.016)	0.008 (0.002, 0.014)	-0.021 (-0.054, 0.013)	0.009 (0.003, 0.015)	-0.023 (-0.056, 0.01)	0.008 (0.002, 0.015)
Walk/light loads at work / inactive in leisure	-0.045 (-0.071, -0.018)	0.011 (0.005, 0.016)	-0.037 (-0.063, -0.011)	0.010 (0.005, 0.015)	-0.034 (-0.060, -0.008)	0.010 (0.005, 0.015)	-0.033 (-0.059, -0.007)	0.010 (0.005, 0.015)
Walk/light loads at work / active in leisure	-0.013 (-0.041, 0.015)	0.010 (0.004, 0.015)	-0.009 (-0.036, 0.019)	0.010 (0.004, 0.015)	-0.005 (-0.033, 0.022)	0.010 (0.005, 0.016)	-0.005 (-0.033, 0.023)	0.010 (0.004, 0.015)
Heavy loads at work / inactive in leisure	-0.118 (-0.185, -0.051)	0.027 (0.015, 0.040)	-0.108 (-0.175, -0.041)	0.026 (0.013, 0.039)	-0.098 (-0.165, -0.032)	0.027 (0.015, 0.040)	-0.090 (-0.157, -0.024)	0.027 (0.014, 0.039)

Heavy loads at work / active in leisure	-0.077 (-0.143, -0.011)	0.019 (0.006, 0.031)	-0.068 (-0.134, -0.002)	0.018 (0.005, 0.031)	-0.068 (-0.134, -0.002)	0.020 (0.008, 0.033)	-0.066 (-0.132, -0.001)	0.019 (0.007, 0.032)

a. **Adjusted for age (centred at baseline), income adequacy, education level, number of children <12 years in the household**
b. **a + adjusted for work stress, work hours, shift type**
c. **b + adjusted for alcohol consumption, smoking status, body mass index**
Note: all models include time as a fixed effect.
